# Normal-to-mildly increased albuminuria predicts the risk for diabetic retinopathy in patients with type 2 diabetes

**DOI:** 10.1038/s41598-017-11906-6

**Published:** 2017-09-18

**Authors:** Min-Kyung Lee, Kyung-Do Han, Jae-Hyuk Lee, Seo-Young Sohn, Oak-Kee Hong, Jee-Sun Jeong, Mee-Kyoung Kim, Ki-Hyun Baek, Ki-Ho Song, Hyuk-Sang Kwon

**Affiliations:** 1 Division of Endocrinology and Metabolism, Department of Internal Medicine, Myongji Hospital, Seonam University College of Medicine, Gyeonggi-do, Republic of Korea; 2 Department of Medical Statistics, College of Medicine, The Catholic University of Korea, Seoul, Republic of Korea; 3 Department of Internal Medicine, College of Medicine, The Catholic University of Korea, Seoul, Republic of Korea; 4 Division of Endocrinology and Metabolism, Department of Internal Medicine, Yeouido St. Mary’s Hospital, College of Medicine, The Catholic University of Korea, Seoul, Republic of Korea

## Abstract

Albuminuria is closely associated with diabetic retinopathy (DR), but the precise role of the albumin-to-creatinine ratio (ACR) in screening for DR remains to be determined. This study aimed to investigate an ACR threshold for predicting DR in patients with type 2 diabetes. A cross-sectional study was conducted on 1,102 type 2 diabetes patients, aged ≥30 years and recruited from the Korea National Health and Nutrition Examination Survey, 2010–2011. Participants were grouped by stage of DR: mild-to-moderate nonproliferative DR (NPDR), severe NPDR, and proliferative diabetic retinopathy (PDR). An early morning spot urine sample was obtained for ACR measurement. ROC curve analysis revealed that the optimal cut-off value of ACR for predicting DR was 2.26 mg/mmol (20 μg/mg). The prevalence of ACR ≥ 2.26 mg/mmol tended to increase with severity of DR. The risk for DR in patients with ACR ≥ 2.26 mg/mmol was higher than in those with ACR < 2.26 mg/mmol. The risk for severe NPDR and PDR also increased at ACR ≥ 2.26 mg/mmol. Normal-to-mildly increased albuminuria (an ACR of 2.26 mg/mmol) may predict the risk for DR development and progression in patients with type 2 diabetes.

## Introduction

Diabetic retinopathy (DR) is a common vascular complication of diabetes, and a leading cause of new-onset blindness^1^. DR progresses from mild nonproliferative abnormalities to moderate and severe nonproliferative DR (NPDR), and to proliferative DR (PDR), which is characterized by gradual alterations in the retinal microvasculature leading to increased vascular permeability, retinal nonperfusion, and pathological intraocular proliferation of retinal vessels^2^. Macular edema, characterized by retinal thickening from leaky blood vessels, can develop at all stages of retinopathy^2^. This complication is highly prevalent^3^ and places a significant burden on society if left untreated^4^; therefore, early detection and identification of the risks for DR are important.

Albuminuria is a known clinical marker of kidney damage. The American Diabetes Association (ADA) and the National Kidney Foundation (NKF) guidelines define microalbuminuria by an albumin-to-creatinine ratio (ACR) of 3.39–33.9 mg/mmol (30–300 μg/mg)^5,6^. Recently, the Kidney Disease: Improving Global Outcomes (*KDIGO*) Clinical Practice Guidelines divided ACR into three categories based on practical considerations, with ACR < 3.39 mg/mmol defined as indicating normal-to-mildly increased albuminuria^7^. High-normal albuminuria is closely associated not only with diabetic kidney disease (DKD)^8,9^, but also with diabetic vascular complications such as cardiovascular disease in patients with type 2 diabetes^10,11^. Clinical studies have reported that albuminuria is associated with DR^12–15^, and albuminuria has an impact on predicting the risk for the development and progression of DR in type 2 diabetes patients. However, the precise role of normal-to-mildly increased albuminuria in screening for DR remains to be determined.

Here, we conducted a cross-sectional study to evaluate the associations between ACR and severity of DR in type 2 diabetes patients grouped by stage of DR. In addition, we determined an ACR threshold for predicting the risk for DR.

## Results

### Characteristics of the study population

The study included a total of 1,102 participants (541 men and 561 women) with type 2 diabetes from the KNHANES. Patients were divided into four groups according to DR stage: 903 patients had no DR, and of the 199 patients with DR, 148 had mild-to-moderate NPDR, 14 had severe NPDR, and 37 had PDR. The baseline clinical characteristics of these groups are displayed in Table 1. Aside from the DR stage, nine patients had CSME: four from the mild-to-moderate NPDR group and five from the PDR group (data not shown). Duration of diabetes, HbA1c level, and FPG increased with NPDR stage (*P* < 0.0001). On the other hand, BMI, waist circumference, and hemoglobin level decreased with NPDR stage (*P* < 0.0001). The prevalence (%) of insulin therapy was higher in more advanced stages of DR. Sex, total cholesterol (TC), and smoking history were not significantly different among the groups (Table 1).

**Table 1:** Baseline characteristics of the study population according to DR stage.

	No DR (n = 903)	Mild to mod NPDR (n = 148)	Severe NPDR (n = 14)	PDR (n = 37)	P value
Age, y	57.96 ± 0.53	60.47 ± 1.06	59.18 ± 2.5	63.14 ± 2.2	0.0512
Sex (male), %	46.4(1.89)	37.61(4.97)	46.35 (15)	49.91(10.48)	0.3502
Duration of diabetes, y	3.48 ± 0.21	9.28 ± 0.75	14.9 ± 3.23	16.3 ± 1.72	<0.0001
Current smoker, %	24.4(1.93)	23.61(4.94)	60.6(14.66)	22.17(8.75)	0.0999
Insulin therapy, %	4.1(0.9)	12.5(3.0)	13.4(10.4)	28.4(9.4)	<0.0001
Oral hypoglycemic agent, %	44.2(2.0)	84.2(4.1)	87.1(7.8)	85.7(6.5)	<0.0001
BMI, kg/m²	25.51 ± 0.15	24.35 ± 0.32	23.96 ± 1.02	23.32 ± 0.51	<0.0001
Waist circumference, cm	88.02 ± 0.39	86.52 ± 0.95	84.79 ± 2.42	83.33 ± 1.27	0.0021
SBP, mmHg	126.48 ± 0.82	132.65 ± 2.22	138.33 ± 9.68	130.43 ± 4.75	0.0337
DBP, mmHg	78.09 ± 0.48	76.98 ± 1.21	77.68 ± 3.35	71.19 ± 1.91	0.0067
Hemoglobin, g/dL	14.36 ± 0.06	14 ± 0.21	13.36 ± 0.32	13.38 ± 0.27	<0.0001
Hemoglobin A1c, %	7.17 ± 0.06	7.94 ± 0.16	8.71 ± 0.42	8.13 ± 0.39	<0.0001
FPG, mg/dL	135.5 ± 1.8	153.75 ± 3.79	172.91 ± 12.31	158.31 ± 10.76	<0.0001
Total cholesterol, mg/dL	193.61 ± 2.05	183.06 ± 4.25	191.68 ± 12.57	169.75 ± 6.8	0.0029
Triglyceride, mg/dL	151.72 (144.33–59.49)	146.87 (132.43–162.9)	174.36 (126.15–241)	114.98 (76.23–173.42)	0.4145
HDL cholesterol, mg/dL	47.28 ± 0.44	47.99 ± 1.37	46.21 ± 4.08	47.4 ± 3.81	0.9472
LDL cholesterol, mg/dL	110.66 ± 1.76	102.47 ± 3.54	109.83 ± 11.78	92.13 ± 4.4	0.0011
Estimated GFR, mL/min/1.73 m^2^	88.86 ± 0.77	83.23 ± 2.34	92.14 ± 8.42	80.98 ± 4.65	0.0423
ACR, mg/mmol^*^	1.12 (1.113–1.126)	1.156 (1.136–1.177)	1.242 (1.147–1.345)	1.2 (1.163–1.238)	<0.0001

Data are presented as mean ± standard error (SE) or proportion (SE).

*Geometric mean (95% CI).

DR, diabetic retinopathy; NPDR, mild-to-moderate nonproliferative; PDR, proliferative diabetic retinopathy; BMI, body mass index; SBP, systolic blood pressure; DBP, diastolic blood pressure; FPG, fasting plasma glucose; HDL, high density-lipoprotein; LDL, low-density lipoprotein; GFR, glomerular filtration; ACR, albumin-to-creatinine ratio.

### ACR levels at different DR stages

ACR levels were significantly different among the DR stages (*P* < 0.0001). The geometric mean of ACR was 1.12 (1.113–1.126) μg/mg in patients without DR, 1.156 (1.136–1.177) μg/mg in those with mild-to-moderate DR, 1.242 (1.147–1.345) μg/mg in those with severe NPDR, and 1.2 (1.163–1.238) μg/mg in those with PDR (Table 1). There was a tendency toward an increase in ACR with severity of DR (*P* for trend < 0.0001).

### ACR as an independent risk factor for DR

A multiple regression model was used to evaluate multivariate-adjusted ORs of DR. Univariate analyses revealed that age, duration of diabetes, insulin therapy, BMI, systolic BP (SBP), hemoglobin, HbA1c, TC, low-density lipoprotein (LDL), eGFR, and ACR were significant risk factors for DR at the level of *P* < 0.20 (Table 2). After performing multivariate regression analysis, we found that age, duration of diabetes, insulin therapy, SBP, HbA1c, and ACR were significantly associated with DR (Table 2). ACR was an independent risk factor for DR in this population (OR = 1.019; 95% CI: 1.006–1.031; *P* = 0.0041).

**Table 2:** Univariate and multivariate analysis of risk factors for DR.

	Unadjusted OR	(95% CI)	P-value	Adjusted OR	(95% CI)	P-value
Age, y	1.02	(1.005–1.035)	0.0101	0.965	(0.94–0.99)	0.0069
Sex (male), %	0.769	(0.511–1.157)	0.2074			
Duration of diabetes, y	1.134	(1.103–1.165)	<0.0001	1.095	(1.054–1.138)	<0.0001
Current smoker, %	1.083	(0.681–1.721)	0.737			
Insulin therapy, %	6.914	(4.037–11.841)	<0.0001	6.539	(3.031–14.105)	<0.0001
Oral hypoglycemic agent, %	4.109	(2.124–7.949)	<0.0001	1.442	(0.579–3.591)	0.4321
BMI, kg/m²	0.888	(0.841–0.938)	<0.0001	0.922	(0.856–0.993)	0.0312
Waist circumflex, cm	0.975	(0.955–0.995)	0.0148			
SBP, mmHg	1.019	(1.007–1.03)	0.0015	1.025	(1.008–1.043)	0.0037
DBP, mmHg	0.985	(0.966–1.003)	0.1049			
Hemoglobin, g/dL	0.837	(0.747–0.937)	0.002	0.911	(0.786–1.056)	0.2175
Hemoglobin A1c, %	1.433	(1.235–1.663)	<0.0001	1.454	(1.241–1.703)	<0.0001
FPG, mg/dL	1.009	(1.005–1.014)	<0.0001			
Total cholesterol, mg/dL	0.993	(0.989–0.998)	0.0063	0.998	(0.982–1.015)	0.8242
Triglyceride, mg/dL	0.999	(0.998–1.001)	0.4316			
HDL cholesterol, mg/dL	1.004	(0.985–1.023)	0.6971			
LDL cholesterol, mg/dL	0.993	(0.988–0.999)	0.0124	1.001	(0.984–1.018)	0.9071
Estimated GFR, mL/min/1.73 m^2^	0.986	(0.975–0.996)	0.0082	0.987	(0.973–1.001)	0.064
ACR, mg/mmol	1.025	(1.013–1.038)	<0.0001	1.019	(1.005–1.033)	0.0088

DR, diabetic retinopathy; BMI, body mass index; SBP, systolic blood pressure; DBP, diastolic blood pressure; FPG, fasting plasma glucose; HDL, high density-lipoprotein; LDL, low-density lipoprotein; GFR, glomerular filtration; ACR, albumin-to-creatinine ratio; CI, confidence interval; OR, odds ratio.

### ACR threshold for predicting DR

**Figure 1:**
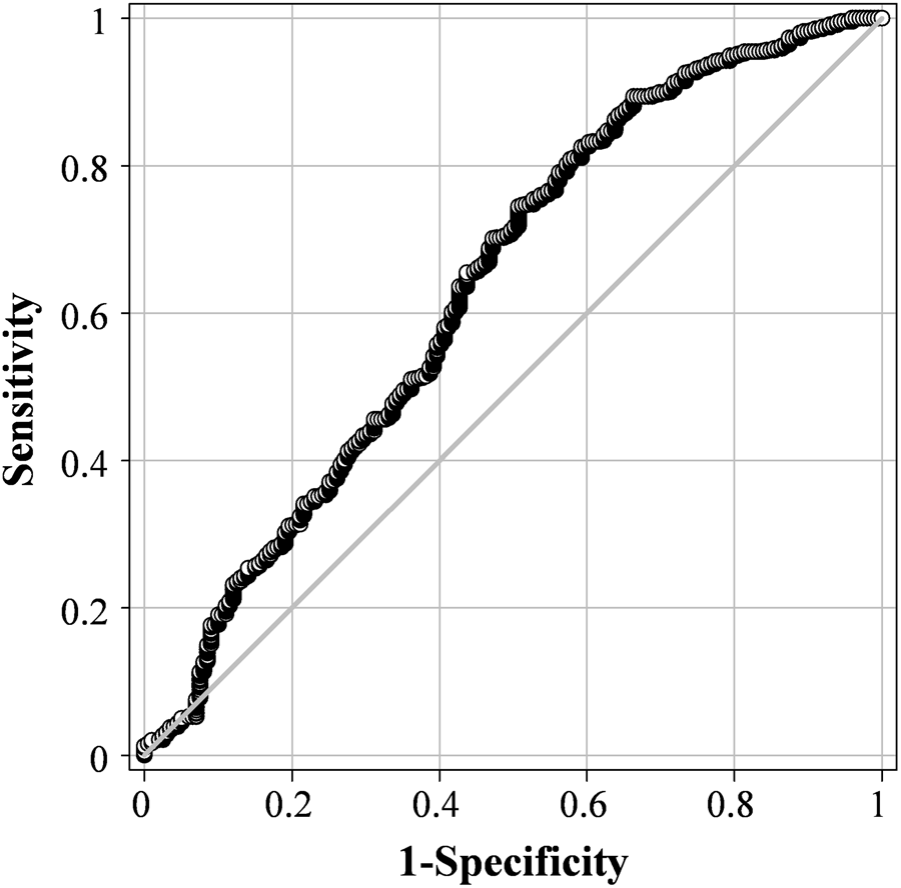
ROC curve of ACR for predicting DR. The AUC for an ACR of 2.26 mg/mmol was 0.634 (95% CI = 0.605–0.663; *P* < 0.0001).

ROC curve analysis was performed to determine the ACR threshold for predicting the risk for DR (Fig. 1). The optimal ACR cut-off value was 2.26 mg/mmol for DR and 0.634 for AUC (95% CI: 0.605−0.663; *P* < 0.0001). The sensitivity, specificity, positive LR, and negative LR were 49.2, 74.1, 1.93, and 0.68, respectively (Table 3). An ACR of 3.39 mg/mmol, which represents the current cut-off point for microalbuminuria, had a sensitivity of 40.7% and specificity of 81.2% for predicting DR (Table 3).

**Table 3:** Sensitivity and specificity of ACR for predicting DR.

ACR cut-off, mg/mmol (μg/mg)	Sensitivity, %	Specificity, %	Positive LRs	Negative LRs
1.808^16^	52.8	69.4	1.73	0.68
2.034^18^	49.7	71.7	1.75	0.7
2.26^20^ *	49.2	74.1	1.93	0.68
2.486^22^	47.2	75.1	1.9	0.7
2.712^24^	44.2	77.2	1.92	0.72
2.938^26^	43.7	78.7	2.07	0.71
3.164^28^	42.2	80.2	2.15	0.72
3.39^30^^†^	40.7	81.2	2.15	0.73
3.616^32^	40.7	82.2	2.25	0.72

*The best cut-off point. ^†^Current cut-off point for microalbuminuria.

ACR, albumin-to-creatinine ratio; DR, diabetic retinopathy; LR, likelihood ratio.

**Figure 2:**
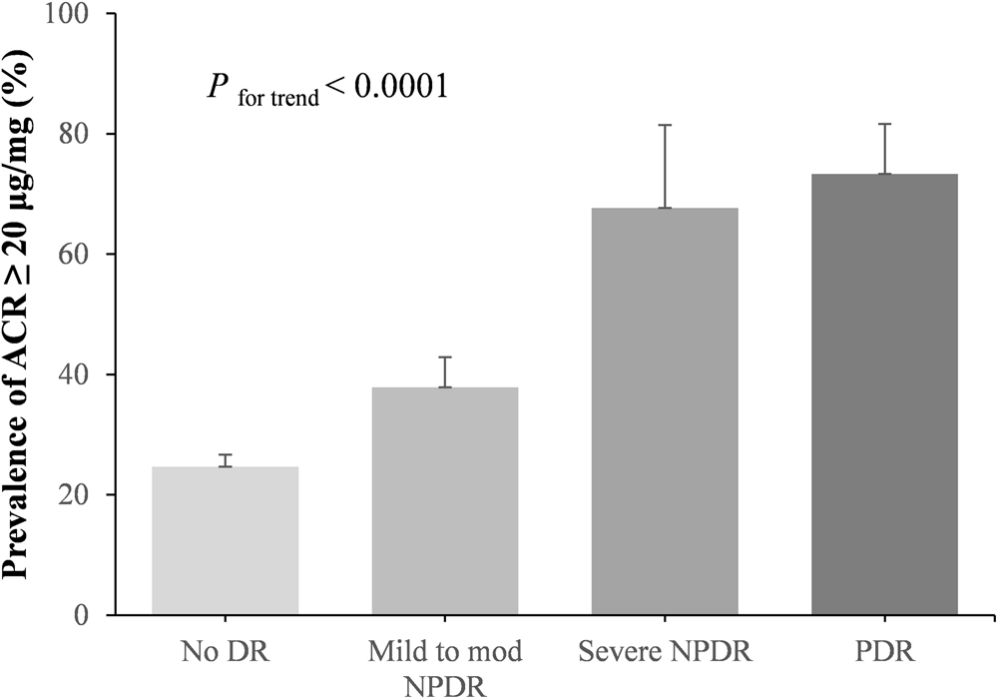
Distribution of patients with ACR ≥ 2.26 mg/mmol at different DR stages. *P* for trend < 0.0001.

Figure 2 presents the distribution of patients with ACR ≥ 2.26 mg/mmol at different DR stages. The prevalence of ACR ≥ 2.26 mg/mmol was 24.7% in patients without DR, 37.8% in those with mild-to-moderate NPDR, 67.68% in those with severe NPDR, and 73.31% in those with PDR, which tended to increase with severity of DR (*P* for trend < 0.0001).

Patients were divided into two groups based on an ACR of 2.26 mg/mmol. Table 4 shows the association between the ACR cut-off level and DR (Table 4). In the univariate analysis, the risk for DR in patients with ACR ≥ 2.26 mg/mmol was significantly higher than in those with ACR < 2.26 mg/mmol (OR = 2.489; 95% CI: 1.695−3.654; *P* < 0.0001). Additionally, the risk for severe NPDR and PDR also increased at ACR ≥ 2.26 mg/mmol (OR = 7.657; 95% CI: 3.631−16.147; *P* < 0.0001). In the multivariable analyses, after adjusting for age and sex (model 1), the risk of DR was significantly increased. In model 2, after adjusting for age, sex, duration of diabetes, insulin therapy, SBP, BMI, HbA1c, and eGFR, the results were similar. Patients with severe NPDR, PDR and CSME showed the same results (Table 4).

**Table 4:** The risk of DR at ACR ≥ 2.26 mg/mmol.

	Crude OR (95% CI)	P-value	Model 1 OR (95% CI)	P-value	Model 2 OR (95% CI)	P-value
All DR stages (n = 199)						
<2.26 (n = 101)	1		1		1	
≥2.26 (n = 98)	2.489 (1.695–3.654)	<0.0001	2.408 (1.634–3.55)	<0.0001	1.722 (1.084–2.734)	<0.0001
Severe NPDR + PDR (n = 51)						
<2.26 (n = 17)	1		1		1	
≥2.26 (n = 34)	7.657 (3.631–16.147)	<0.0001	7.291 (3.391–15.678)	<0.0001	9.51 (3.28–27.575)	<0.0001
Severe NPDR + PDR + CSME (n = 55)						
<2.26 (n = 20)	1		1		1	
≥2.26 (n = 35)	8.117 (3.467–19.005)	<0.0001	7.552 (3.124–18.257)	<0.0001	10.103 (3.294–30.992)	<0.0001

Model 1 is adjusted for age, sex. Model 2 is adjusted for age, sex, duration of diabetes, insulin therapy, SBP, BMI, HbA1c, and eGFR.

DR, diabetic retinopathy; NPDR, mild-to-moderate nonproliferative; PDR, proliferative diabetic retinopathy; BMI, body mass index; SBP, systolic blood pressure; GFR, glomerular filtration; ACR, albumin-to-creatinine ratio; CI, confidence interval; OR, odds ratio; CSME, clinically significant macular edema.

## Discussion

In the present study, we found that ACR level was independently associated with DR and its severity in patients with type 2 diabetes. ACR levels differed according to the stage of DR, and tended to increase with severity of DR. Moreover, the study showed that normal-to-mildly increased albuminuria was a predictor of the risk for DR. ROC curve analysis revealed that the optimal ACR cut-off value for predicting the risk for DR was 2.26 mg/mmol. Logistic regression analysis revealed that the risk for DR or VTDR significantly increased at ACR ≥ 2.26 mg/mmol, suggesting that this ACR threshold may predict the risk for DR development and progression.

The association between albuminuria and DR has already been documented in several studies of type 2 diabetes patients^15–17^. One potential explanation for this association is that microalbuminuria may represent a state of generalized vascular dysfunction^18^. DR and DKD are both microvascular complications of diabetes, and are characterized by similar pathophysiological mechanisms^19^. The microvascular changes in both the retina and glomerulus are thought to be initiated by chronic hyperglycemia, followed by the progressive narrowing and eventual occlusion of the vascular lumina. In the retina, diabetes induces programmed cell death of Müller and ganglion cells^20^, as well as the loss of endothelial cells in capillaries and the loss of pericytes; this leads to progression of DR. In the glomerulus, widespread capillary occlusion and podocyte loss cause urinary protein loss and a decline in renal function. In this study, ACR levels were higher at more advanced stages of DR. This supports the hypothesis that the progression of DR involves the same mechanisms as vascular dysfunction in DKD.

Improved DR screening rates are associated with less frequent visual impairment among patients with diabetes^21^. Therefore, early detection of DR risk factors is critical. ACR widely used to diagnose DKD^22^, but studies evaluating the use of ACR in the screening process for DR are limited. In this study, we found that ACR was an independent risk factor for DR, and the risk for DR significantly increased at an ACR ≥ 2.26 mg/mmol. In several studies, the prevalence of DR in normal albuminuria has been reported to be 10−20%^14,23,24^. Consistent with our finding, a recent study reported that normal-to-mildly increased albuminuria may be a strong predictor for DR^3^, indicating that the risk for DR gradually increases with ACR levels below the microalbuminuria threshold^25^. Additionally, we found that the risk for more advanced stages of DR was higher based on an ACR of 2.26 mg/mmol. VTDR, including severe NPDR, PDR, and CSME, may result in rapid vision loss if left untreated. An ACR of 2.26 mg/mmol may predict more advanced stages of DR, and informs diagnostic criteria for treatment to prevent vision loss.

Several studies have indicated that normal-to-mildly increased albuminuria (<3.39 mg/mmol) is associated with a higher risk of diabetic complications, such as coronary artery disease, heart failure, and atherosclerosis^26–28^. It has been reported that normal-to-mildly increased albuminuria may be a risk factor for cardiovascular disease, in patients with and without diabetes^29^. Our study documented that patients with normal-to-mildly increased albuminuria had a high risk for DR and VTDR after adjusting for age, sex, duration of diabetes, SBP, BMI, HbA1c, and eGFR. This suggests that normal-to-mildly increased albuminuria may predict the development of DR and progression of VTDR independent of several cardiovascular risk factors.

Our study did not exclude patients who were using angiotensin-converting enzyme inhibitor (ACEI) or angiotensin II receptor blocker (ARB) drugs to evaluate the association between albuminuria and DR. Multiple studies have shown that the use of ACEI or ARB drugs decreases ACR levels. It is unclear whether this is due to the antihypertensive or antiproteinuric effects of the drugs. On the other hand, several studies have shown that renin-angiotensin system (RAS) inhibitors reduce the risk for the development and progression of DR and increase its regression^30^. Intensive BP control with RAS inhibitors can be expected to reduce ACR levels and prevent progression to DR in patients with normal-to-mildly increased albuminuria. More large-scale randomized controlled trials are needed to further clarify the association between ACR and DR in patients using ACEI or ARB drugs.

This study had a cross-sectional design using data from KNHANES. The study exhibits several strengths. First, this study used a general population-based data set; thus, we could reduce selection bias, unlike when using hospital data. Second, the study showed that ACR levels were not only associated with DR, but also increased with the severity of DR, by dividing diabetes patients by DR stage. Third, we used a ROC curve to determine the cut-off value of ACR to predict the risk of DR, and there was a significant difference in the risk of DR between the two groups divided based on this cut-off value.

This study used data from the KNHANES had a cross-sectional design. The study exhibits several strengths. First, this study used a general population-based data set; thus, we could reduce selection bias, unlike when using hospital data. Second, the study showed that ACR levels were not only associated with DR, but also increased with the severity of DR by dividing diabetes patients by DR stage. Third, we used a ROC curve to determine the cut-off value of ACR to predict the risk for DR, and there was a significant difference in the risk for DR between the two groups divided based on this cut-off value.

This study also had several limitations. First, we could not infer any causal relationships between ACR and DR due to the cross-sectional design. To our knowledge, there is no clinical trial specifically designed to evaluate the effect of an increase or reduction of ACR on DR progression; this should be confirmed in additional case-control trials. Second, we evaluated the urinary albumin excretion (UAE) using a single early morning urine sample rather than 24-h urine or multiple samples. Although ACR in a single morning urine sample is less precise, the use of spot samples for urinary ACR is recommended in the clinical practice, because this test can be easily performed in the outpatient clinic, and the results correlate well with those of 24-h UAE and multiple samples^31,32^. Third, ACR values may vary based on urine creatinine excretion^33^; further studies are needed to determine appropriate ACR thresholds for different age/sex groups.

In conclusion, the present study showed that ACR level was associated with DR and its severity in patients with type 2 diabetes. Moreover, the study suggests that an ACR of 2.26 mg/mmol is the optimal cut-off level for predicting the risk for DR. Therefore, normal-to-mildly increased albuminuria in type 2 diabetes patients should not be overlooked by clinicians and requires close monitoring for early detection of DR.

## Methods

### Study population and design

The data for this study were taken from the Korea National Health and Nutrition Examination Survey (KNHANES), 2010−2011. The KNHANES is a nationally representative cross-sectional survey conducted annually by the Korea Centers for Disease Control and Prevention (KCDC). Information on socioeconomic status, health-related behaviors, quality of life, healthcare utilization, anthropometric measures, biochemical and clinical profiles of non-communicable diseases, and dietary intake is collected by trained investigators. The data from the KNHANES provide statistics informing health-related policies in Korea, and are also used for studies on risk factors and diseases^34^. All participants signed an informed consent form and this survey was approved by the Institutional Review Board of the Catholic University of Korea (IRB No. SC14EISE0108). This study was conducted according to the Helsinki Declaration-based ethical principles for medical research involving human subjects.

This study included a total of 12,859 adults aged ≥30 years. Diabetes was confirmed by a fasting plasma glucose (FPG) level ≥126 mg/dL, or when a participant was receiving insulin or oral hypoglycemic agents, or by a self-reported history of physician diagnosis. We excluded 11,423 participants who did not have diabetes, and 323 who with missing data related to diabetes. Ultimately, 1,102 participants (541 men and 561 women) with diabetes were included in the analysis.

### Clinical information and laboratory analysis

The physical examination was performed by measuring height, weight, and waist and hip circumferences according to standardized methods. Body mass index (BMI) was calculated by dividing weight (kg) by the square of height (m^2^). Blood pressure (BP) was measured with the participant in a seated position using a Baumanometer® Desk (model 0320; W. A. Baum Co., Inc., Copiague, NY, USA). BP was measured in triplicates, and the mean value of the second and third measurements was used for the analysis. Blood and urine samples were obtained on the morning after an overnight fast of at least 8 hours. FPG, cholesterol, and urine albumin were measured with a Hitachi automatic analyzer 7600 (Hitachi Ltd., Tokyo, Japan). Glycated hemoglobin (HbA1c) was measured with an HLC-723G7 (Tosoh, Tokyo, Japan). The estimated glomerular filtration rate (eGFR) was calculated using the Modification of Diet in Renal Disease study equation^35^. ACR (mg/mmol) was calculated as the spot urine albumin concentration (mg/L) divided by the spot urine creatinine (mmol/L)^31^.

### Ophthalmic examination and definition of diabetic retinopathy

Participants underwent ocular examinations, including fundus photographs. A digital non-mydriatic fundus camera (TRC-NW6S; Topcon, Tokyo, Japan) and a Nikon D-80 digital camera (Nikon, Tokyo, Japan) were used to obtain images of the digital fundus. For each participant, one 45° non-mydriatic digital retinal image centered on the fovea was taken per eye (two images per person)^36^. For participants with a history of DM, a random blood glucose level of ≥200 mg/dL, and/or suspected DR indicated on non-mydriatic fundus photography, seven standard field photographs were obtained from each eye after pharmacological pupil dilatation, as per the Early Treatment for Diabetic Retinopathy Study (ETDRS) protocol^37^. DR was identified by the presence of any characteristic lesion determined by the ETDRS severity scale^1^: microaneurysm^2^, dot hemorrhages^3^, hard exudates^4^, cotton wool spots^5^, venous beading^6^, intraretinal microvascular abnormalities^7^, retinal new vessels^8^, vitreous hemorrhage^9^, fibrous proliferation^10^, tractional retinal detachments^11^, previous laser therapy, and^12^ phthisis bulbi^38^.

In the current study, DR was classified as no DR, mild-to-moderate NPDR (1−4), severe NPDR (5−6), or PDR (7−12) based on international clinical diabetic retinopathy and macular edema disease severity scales, depending on the presence of specific DR features^39^. Clinically significant macular edema (CSME), defined according to the ETDRS criteria, can occur at any stage of DR independent of any other features^40^. Vision-threatening diabetic retinopathy (VTDR) was defined as the presence of severe NPDR, proliferative retinopathy, or CSME^41^. The quality of the survey was verified by the Epidemiologic Survey Committee of the Korean Ophthalmologic Society.

### Statistical analysis

All statistical analyses were performed using SAS software (ver. 9.3; SAS Institute, Cary, NC, USA). The KNHANES data used multiple complex survey designs, such as stratification, multiple stages of cluster selection, and oversampling to obtain a representative sample of the target population^42^. Patient characteristics were compared according to the stage of DR by analysis of variance (ANOVA) for continuous variables and Pearson’s chi-square test for categorical variables. Data are presented as mean ± standard error (SE) for continuous variables or proportion (SE) for categorical variables. Geometric means were used for highly skewed data [95% confidence interval (CI)]. A general linear model was used to test for a linear trend in ACR by stage of DR as a continuous variable. Multiple logistic regression analysis was used to identify the factors that were independently associated with DR. Variables that were significant at the P < 0.20 level in the univariate analyses were included in multivariate forward-stepwise regression models to calculate adjusted odds ratios (ORs) and their 95% CIs. Analyzing the area under the curve (AUC) of the plotted receiver operating characteristics (ROC) curve represents the optimal cut-off level of ACR by assessing the ability to predict the risk for DR^43^. The ROC curve was calculated to evaluate the sensitivity and specificity of ACR for DR, and the Youden index was estimated to determine optimal cut-off values. We calculated the positive and negative likelihood ratios (LRs). Logistic regression was also used to evaluate the associations between ACR and DR stages in three different models. All reported *P* values were two-sided. Significance was set at *P* < 0.05, and CIs were calculated at the 95% level.
