# Publisher Correction: Normal-to-mildly increased albuminuria predicts the risk for diabetic retinopathy in patients with type 2 diabetes

**DOI:** 10.1038/s41598-018-24854-6

**Published:** 2018-04-25

**Authors:** Min-Kyung Lee, Kyung-Do Han, Jae-Hyuk Lee, Seo-Young Sohn, Oak-Kee Hong, Jee-Sun Jeong, Mee-Kyoung Kim, Ki-Hyun Baek, Ki-Ho Song, Hyuk-Sang Kwon

**Affiliations:** 1 Division of Endocrinology and Metabolism, Department of Internal Medicine, Myongji Hospital, Seonam University College of Medicine, Gyeonggi-do, Republic of Korea; 2 Department of Medical Statistics, College of Medicine, The Catholic University of Korea, Seoul, Republic of Korea; 3 Department of Internal Medicine, College of Medicine, The Catholic University of Korea, Seoul, Republic of Korea; 4 Division of Endocrinology and Metabolism, Department of Internal Medicine, Yeouido St. Mary’s Hospital, College of Medicine, The Catholic University of Korea, Seoul, Republic of Korea

Correction to: *Scientific Reports* 10.1038/s41598-017-11906-6, published online 18 September 2017

This Article contains typographical errors in the first column of Table 3, titled ‘ACR cut-off, mg/mmol (µg/mg)’, where references were inadvertently inserted in place of text. The correct Table 3 appears below as Table 1.

**Table 1:** Sensitivity and specificity of ACR for predicting DR.

ACR cut-off, mg/mmol (μg/mg)	Sensitivity, %	Specificity, %	Positive LRs	Negative LRs
1.808 (16)	52.8	69.4	1.73	0.68
2.034 (18)	49.7	71.7	1.75	0.7
2.26 (20)*	49.2	74.1	1.93	0.68
2.486 (22)	47.2	75.1	1.9	0.7
2.712 (24)	44.2	77.2	1.92	0.72
2.938 (26)	43.7	78.7	2.07	0.71
3.164 (28)	42.2	80.2	2.15	0.72
3.39 (30)^†^	40.7	81.2	2.15	0.73
3.616 (32)	40.7	82.2	2.25	0.72

^*^The best cut-off point. ^†^Current cut-off point for microalbuminuria.

ACR, albumin-to-creatinine ratio; DR, diabetic retinopathy; LR, likelihood ratio.

